# The long intergenic noncoding RNA GAS5 reduces cisplatin-resistance in non-small cell lung cancer through the miR-217/LHPP axis

**DOI:** 10.18632/aging.202352

**Published:** 2021-01-08

**Authors:** Xuhui Yang, Lifei Meng, Yuang Zhong, Fengqing Hu, Lei Wang, Mingsong Wang

**Affiliations:** 1 Department of Thoracic Surgery, Shanghai Ninth People’s Hospital, Shanghai JiaoTong University School of Medicine, Shanghai, China; 2 Department of Cardiothoracic Surgery, Xinhua Hospital, Shanghai JiaoTong University School of Medicine, Shanghai, China; 3 Department of Thoracic Surgery, Ningbo First Hospital, Ningbo, China

**Keywords:** non-small cell lung cancer, miR-217, LHPP

## Abstract

Long noncoding RNAs (lncRNAs) are known to exert their effects to tumor progression. In this study, the role of the lncRNA GAS5 (growth arrest specific 5) was confirmed in reducing non-small cell lung cancer (NSCLC) cisplatin (DDP) resistance. In NSCLC tissue samples, GAS5 expression decreased significantly. Low GAS5 levels were positively correlated with NSCLC characteristics including TNM, tumor size and lymphatic metastasis. Functionally, GAS5 significantly reduced NSCLC/DDP cell migration, invasion and epithelial-mesenchymal transition (EMT) progression *in vitro*. *In vivo*, GAS5 upregulation inhibited remarkably NSCLC/DDP cell tumor growth. Mechanism analysis suggested that GAS5 was a molecular sponge of miR-217, inhibiting the expression of phospholysine phosphohistidine inorganic pyrophosphate phosphatase (LHPP). In conclusion, this study reveals that the GAS5/miR-217/LHPP pathway reduces NSCLC cisplatin resistance and that LHPP may serve as a potential therapeutic target for NSCLC cisplatin resistance.

## INTRODUCTION

The morbidity and death rate of Non-small-cell lung cancer (NSCLC) are the first in the world. Although advances in medical oncology, the mortality rate for advanced NSCLC is still very high [1]. Even after receiving standard platinum-based chemo-treatment, these patients usually show a slight improvement, causing a limited increase in poor survival rates [2]. Numerous factors such as genetic causes of resistance to cisplatin (DDP) – based therapy [3] is a major reason why treated NSCLC patients do not always properly respond to therapy. Therefore, it is urgent to study the mechanisms behind chemotherapy sensitivity in NSCLC patients.

There is a great deal of evidence showing that lncRNAs serve as important regulators in cancer [4] where they promote or suppress tumors [5]. The Growth Arrest-Specific 5 lncRNA (lncGAS5, GAS5) serves as an antioncogene linked to many cancers and has been isolated from NIH3T3 cells [6]. GAS5 has shown to be downregulated in cancers [7–12]. Lower levels of GAS5 is a poor prognosis indicator in cancer patients. Downregulation of GAS5 has also been linked to drug-resistance. For example, downregulation of GAS5 causes trastuzumab-resistance in breast cancer [13] and can also reduce Adriamycin sensitivity in gastric cancer [7]. Importantly, GAS5 has been reported to regulate NSCLC cisplatin-resistance [14]. However, how GAS5 reduces NSCLC cisplatin resistance need to further be evaluated.

LHPP, full name was Phospholysine phosphohistidine inorganic pyrophosphate phosphatase and was first reported in 2009 years [15, 16] and restrains human hepatocellular carcinoma (HCC) through the inhibition of PI3K/AKT signaling pathway [17]. Silencing LHPP in cervical cancer promotes cell metastasis, apoptosis and proliferation through AKT [18]. However, the role of LHPP in cisplatin-resistant NSCLC has not yet been identified.

MiRNAs can degrade mRNAs and inhibit protein expression [19]. In cutaneous squamous cell carcinoma, ectopic miR-217 gene expression promotes cell growth, the cell cycle and invasion to inhibit PTRF expression [20]. Jiang *et al* reported that miR-217 enhanced cancer stem cell properties through activating Wnt signaling [21]. Here, we searched for the potential GAS5 target to find miR-217 as a potential candidate on online databases (Starbase, http://starbase.sysu.edu.cn/index.php). Therefore, we proposed that GAS5 may regulate cisplatin-resistance of NSCLC through the miR-217/LHPP pathway in NSCLC. Meanwhile, we predict LHPP as a putative target of miR-217 by online database (TargetScan, http://www.targetscan.org/vert_72/). We showed that GAS5 overexpression reduced NSCLC cell cisplatin-resistance. GAS5 knockdown promoted NSCLC sensitivity to cisplatin. Importantly, LHPP was confirmed as a target molecule of the GAS5/miR-217 signaling pathway. Overall, our findings illustrate the underlying mechanism of GAS5 and LHPP in NSCLC cisplatin-resistance.

## RESULTS

### GAS5 is decreased in NSCLC/DDP cells and is associated with clinicopathologic characteristics

First, we established NSCLC/DDP cell including A549/DDP and H1299/DDP cells and calculated IC_50_ values for cisplatin. The IC_50_ values of cells were both significantly higher than parental cells (Supplementary Figure 1A, 1B). Subsequently, we found that GAS5 expression was reduced in NSCLC/DDP cells (Supplementary Figure 1C, 1D). These results demonstrated that GAS5 may participated in the cisplatin-resistance seen in NSCLC cases.

**Figure 1:**
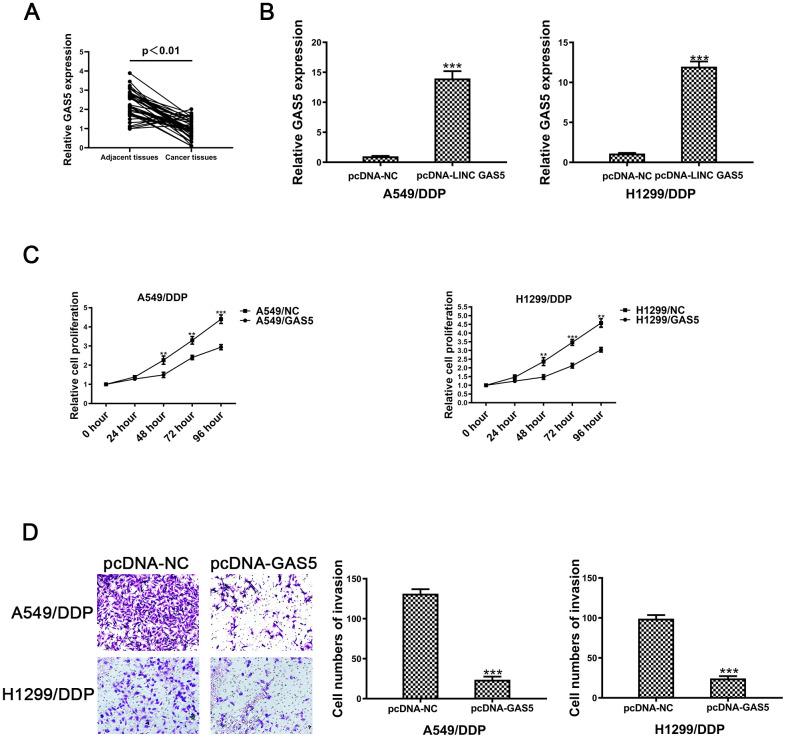
**The lncRNA GAS5 acts as a tumor suppressor in cisplatin-resistant NSCLC cells.** (**A**) Expression of GAS5 in NSCLC and paired adjacent normal tissue samples was determined by qRT-PCR (n=41). (**B**) The expression of GAS5 in A549/DDP and H1299/DDP cells transduced with pcDNA-lncRNA GAS5 or pcDNA-NC was detected by qRT-PCR. (**C**) Cell viability of A549/DDP and H1299/DDP cells transfected with pcDNA-lncRNA GAS5 or pcDNA-NC was determined by CCK-8. (**D**) The invasion of A549/DDP and H1299/DDP cells transfected with pcDNA- lncRNA GAS5 or pcDNA-NC was determined by a transwell assay. *p < 0.05, **p < 0.01, ***p < 0.001. The same experiments were performed at least three times.

Second, relative expression levels of GAS5 were evaluated in 41 pairs of NSCLC and tumor adjacent tissue samples. Figure 1A revealed that GAS5 levels in paired adjacent normal tissues were remarkably higher than levels observed in NSCLC tissues. Expression levels of GAS5 were negatively associated with tumor status progression (Table 1) (p = 0.017^*^), tumor size (p = 0.036^*^) and lymphatic metastasis (p = 0.028^*^) (Table 1). The expression levels of GAS5 were not significantly associated with gender or age. Together, this data revealed that decreased GAS5 expression may be involved in NSCLC progression.

**Table 1:** The association of LINC GAS5 expression in 41 NSCLC patients with clinicopathologic characteristics.

**Characteristics**	**Patients**	**Expression of LinGAS5**		***P*-value**
		**High-LincGAS5**	**Low-LincGAS5**	
**Total**	41	20	21	
**Gender**				0.295
Male	23	9	14	
Female	18	10	8	
**Age(years)**				0.938
≤60	19	8	11	
≥60	22	9	13	
**TNM stage**				0.017^*^
I-II	21	13	8	
III-IV	20	5	15	
**Tumor size**				0.036^*^
<3cm	15	9	6	
>3cm	26	7	19	
**Lymphatic metastasis**				0.028^*^
No	17	10	7	
Yes	24	6	18	

To further confirm the role of GAS5 in NSCLC/DDP cell, A549/DDP and H1299/DDP cells were transduced withpcDNA-GAS5 or pcDNA-NC. Expression of GAS5 was detected by qRT-PCR (Figure 1B). After transfecting pcDNA-GAS5, the IC_50_ value was reduced in NSCLC/DDP cells (Supplementary Figure 1E, 1F). However, when GAS5 was knockdown, the IC_50_ value was increased dramatically (Supplementary Figure 1G, 1H). Cell proliferation was repressed after transfection of pcDNA-GAS5 in DDP cells (Figure 1C). In addition, cell invasion was also inhibited by upregulating GAS5 (Figure 1D). These data revealed that GAS5 overexpression was able to inhibit the proliferation and invasion of cisplatin-resistant NSCLC cells.

### Reciprocal suppression of GAS5 and miR-217 in NSCLC/DDP cells.

**Figure 2:**
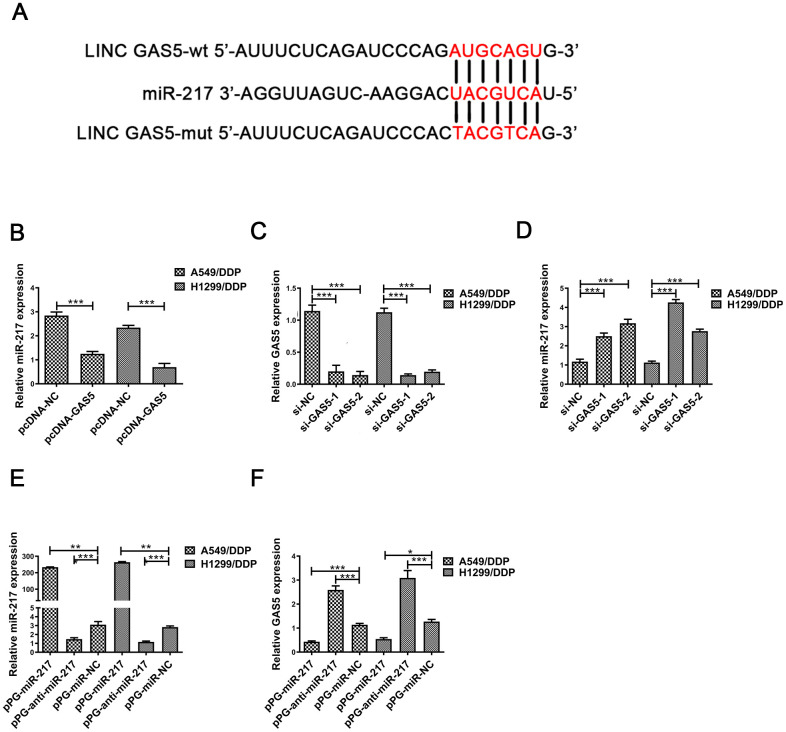
**MiR-217 acts as a target of GAS5.** (**A**) A schematic diagram of the sequence of miR-217 with GAS5 and GAS5 mutated at the putative binding site. (**B**) Relative expression of miR-217 in A549/DDP and H1299/DDP cells transfected with pcDNA- lncRNA GAS5 or pcDNA-NC measured by qRT-PCR. (**C**) The relative expression of GAS5 in A549/DDP and H1299/DDP cells transfected with si-NC, si-GAS5-1 and si-GAS5-2 were detected by qRT-PCR. (**D**) The relative expression of miR-217 in A549/DDP and H1299/DDP cells transfected with si-NC, si-GAS5-1 and si-GAS5-2 was detected by qRT-PCR. (**E**) The relative expression of miR-217 in A549/DDP and H1299/DDP cells transfected with pPG-miR-217, pPG-anti-miR-217 and pPG-miR-NC was quantified by qRT-PCR. (**F**) The relative expression of GAS5 in A549/DDP and H1299/DDP cells transfected with pPG-miR-217, pPG-anti-miR-217 and pPG-miR-NC was detected by qRT-PCR. *p < 0.05, **p < 0.01, ***p < 0.001. The same experiments were performed at least three times.

Many studies have reported that lncRNAs combine competitively with miRNA binding sites to control gene expression [22–24]. Previous studies demonstrated that GAS5 may interact with miRNA to reduce protein expression [25–28]. We hypothesized that GAS5 was able to interact with miRNAs in NSCLC/DDP cells. The same responsive element was found in GAS5 and miR-217 by online databases (Starbase, http://starbase.sysu.edu.cn/index.php). A putative binding site was shown in Figure 2A. Previous studies demonstrated that miR-217 expression was upregulated in many cancers [20, 21, 29]. MiR-217 expression was found to be reduced in pcDNA-GAS5 transfected NSCLC/DDP cells (Figure 2B). Moreover, qRT-PCR was carried out to determine expression levels of miR-217 in si-GAS5-1 or si-GAS5-2 transfected A549 and H1299 cells. As expected, miR-217 expression was increased in si-GAS5-1 or si-GAS5-2 transfected NSCLC/DDP cells (Figure 2D). Importantly, cisplatin-resistance was promoted in NSCLC cells transfected with si-GAS5 (Supplementary Figure 1G, 1H).

To further determine whether miR-217 negatively regulates lncRNA GAS5, A549/DDP and H1299/DDP cells were transduced with pPG-miR-217, pPG-anti-miR-217 or pPG-NC. qRT-PCR results indicated that pPG-anti-miR-217 dramatically decreased miR-217 expression and improved GAS5 expression, whereas pPG-miR-217 significantly promoted miR-217 expression and inhibited GAS5 expression (Figure 2E, 2F).

### GAS5 regulates LHPP by directly binding to miR-217

**Figure 3:**
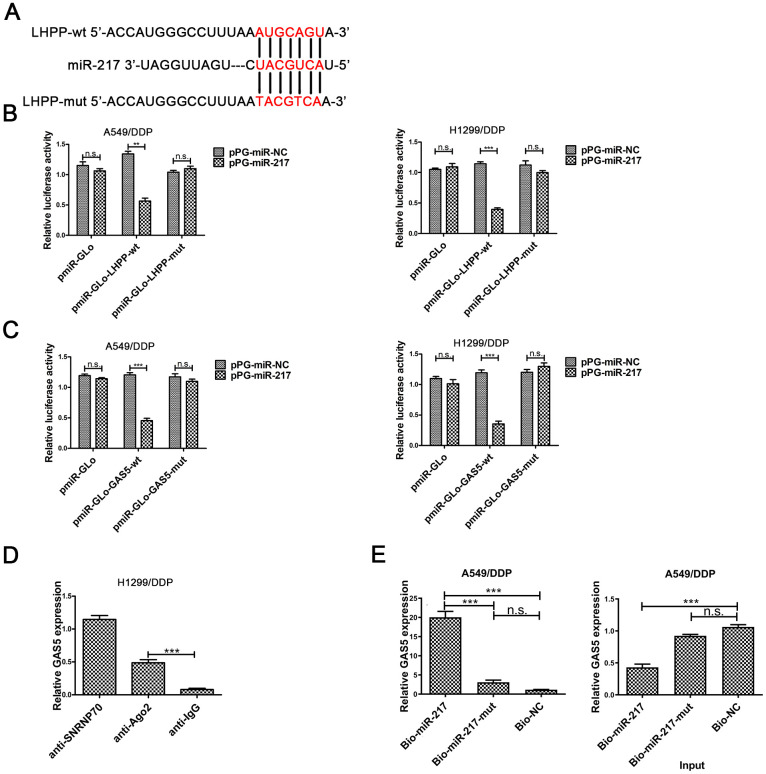
**The underlying mechanism between LHPP, miR-217 and GAS5.** (**A**) A schematic diagram of the miR-217 sequence with LHPP and with LHPP mutated at the putative binding site. (**B**) Luciferase reporter activity in A549/DDP and H1299/DDP cells was measured after co-transfection with pPG-miR-217 (or the empty vector as a control) and the luciferase empty vector (pmiR-GLo), or the vector containing the wild-type LHPP (pmiR-GLo-LHPP-wt) or mutant transcripts (pmiR-GLo-LHPP-mut). (**C**) Luciferase reporter activity in A549/DDP and H1299/DDP cells was measured after co-transfection with pPG-miR-217 (or the empty vector as a control) and the luciferase empty vector (pmiR-GLo), or the vector containing the wild-type GAS5 (pmiR-GLo-GAS5-wt) or mutant transcripts (pmiR-GLo-GAS5-mut). (**D**) The amount of GAS5 bound to SNRNP70 (positive control), Ago 2 or lgG (negative control) was determined by qRT-PCR after RIP in H1299 cells. MiR-217 exert their miRNA-mediated gene silencing function by binding to Ago2, a core component of the RNA-induced silencing complex (RISC). When miR-217 forms a RISC complex, it is wrapped by Ago protein, mainly Ago2. Pulling down the Ago2 protein will pull down the miR-217 bound to it. LncRNA GAS5 bound by RISC will also be pulled down. GAS5 expression levels were measured by qPCR. (**E**) A549/DDP cells were transfected with biotinylated NC (Bio-NC), biotinylated wild-type miR-217 (BiomiR-217) or biotinylated mutant miR-217 (Bio-miR-217-mut), and biotin-based miRNA pull-down assays were conducted after 48 h of transfection. biotinylated wild-type miR-217 can combine lncRNA GAS5. Bead combines biotinylated and lncRNA GAS5 will be pulled down. GAS5 expression levels were measured by qPCR. *p < 0.05, **p < 0.01, ***p < 0.001. The same experiments were performed at least three times.

We predicted putative targets of miR-217 by online database analysis (TargetScan, http://www.targetscan.org/vert_72/).Figure 3A showed the putative binding site between miR-217 and LHPP mRNA. To further verify the reciprocity between the lncRNA, miR-217 and LHPP, we carried out a dual luciferase reporter assay in A549/DDP and H1299/DDP cells to confirm the functionality of the site. Dual luciferase reporter results showed that luciferase activity was significantly decreased in A549/DDP and H1299/DDP cells transfected with pmiR-Glo-LHPP-wt, while there was not change in NSCLC/DDP cells transfected with pmiR-Glo-LHPP-mut (Figure 3B). These results revealed that miR-217 directly bound with LHPP 3’UTR in A549/DDP and H1299/DDP cells.

To further investigate whether the predicted binding site between GAS5 and miR-217, was carried out the dual-luciferase reporter assay in A549/DDP and H1299/DDP cells. Luciferase activity was reduced in pPG-miR-217 + pmiR-Glo-GAS5-wt group, was not changed in pPG-miR-217 + pmiR-Glo or pPG-miR-217 + pmiR-Glo-GAS5-mut (Figure 3C). These results demonstrated the predicted binding site was necessary for the interactional repression of GAS5 and miR-217.

miRNA causes gene silencing by binding to Ago2, which is the core component of the RNA-induced silencing complex (RISC) [30]. The results showed that GAS5 was preferentially enriched in Ago2-containing beads in H1299/DDP cells (Figure 3D). In addition, pull-down was carried out in A549/DDP cells. Pull down results showed that GAS5 could be pulled down by miR-217 (Figure 3E). There was decreased expression of GAS5 in the input samples for A549/DDP cells transfected with biotinylated miR-217. Overexpression of biotinylated miR-217 resulted in decreased expression of GAS5 in A549/DDP cells, which was consistent with our previous findings. These results demonstrated that GAS5 improved LHPP expression by directly combining to the miR-217 binding site.

### GAS5 promotes LHPP expression by competitively interacting with miR-217

**Figure 4:**
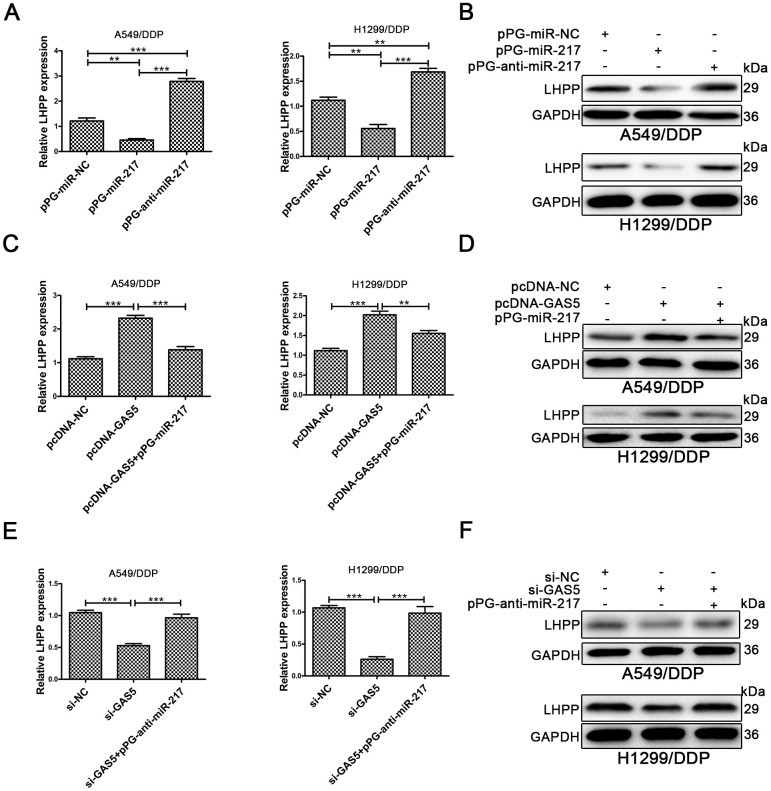
**LHPP is located downstream of miR-217 and l GAS5.** (**A**, **B**) Both qRT-PCR and Western blotting were used to determine the relative mRNA and protein expression of LHPP in A549/DDP and H1299/DDP cells transfected with pPG-miR-NC, pPG-miR-217 and pPG-anti-miR-217. (**C**, **D**) qRT-PCR and Western blotting were used to determine the relative mRNA and protein levels of LHPP in A549/DDP and H1299/DDP cells transfected with pcDNA-NC, pcDNA-GAS5 or pcDNA-GAS5 + pPG-miR-217. (**E**, **F**) qRT-PCR and Western blotting were used to determine the relative mRNA and protein levels of LHPP in A549/DDP and H1299/DDP cells transfected with si-NC, si-GAS5 or si-GAS5 + pPG-anti-miR-217. *p < 0.05, **p < 0.01, ***p < 0.001. The same experiments were performed at least three times.

The LHPP mRNA and protein expression in A549/DDP and H1299/DDP cells was determined by qRT-PCR and Western blotting. LHPP expression levels were increased in A549/DDP and H1299/DDP cells transduced with pPG-anti-miR-217, and decreased in cells with pPG-miR-217 (Figure 4A, 4B, Supplementary Figure 2A). Next, we investigated whether GAS5 could promote LHPP expression by competing with miR-217 in cisplatin-resistant NSCLC cells. Upregulation of GAS5 increased LHPP expression in A549/DDP and H1299/DDP cells, whereas pPG-miR-217 reduced the role of GAS5 (Figure 4C, 4D, Supplementary Figure 2B). GAS5 knockdown in A549/DDP and H1299/DDP cells markedly restrained LHPP while pPG-anti-miR-217 reversed these effects (Figure 4E, 4F, Supplementary Figure 2C). These results demonstrated that GAS5 increased LHPP expression by binding to miR-217.

### LHPP inhibits NSCLC cell EMT progression, proliferation and invasion in NSCLC/DDP cells

**Figure 5:**
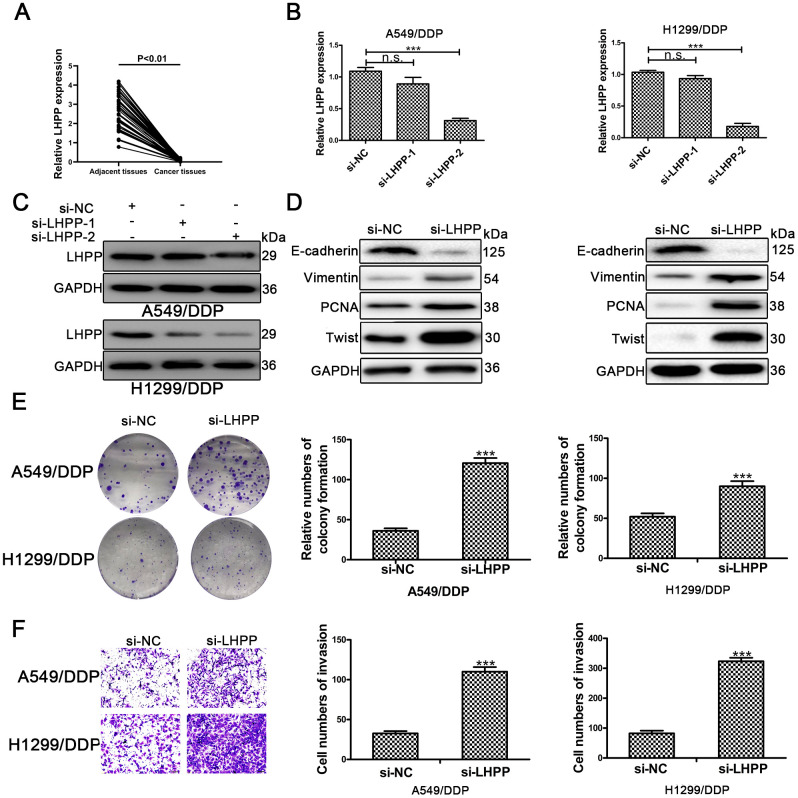
**LHPP serves as a tumor suppressor in cisplatin-resistant NSCLC cells.** (**A**) The relative mRNA expression of LHPP in NSCLC tissues and their paired adjacent normal tissues was determined by qRT-PCR. (**B**) The relative mRNA expression of LHPP in A549/DDP and H1299/DDP cells transfected with si-NC, si-LHPP-1 and si-LHPP-2 was measured by qRT-PCR. (**C**) The relative protein expression of LHPP in A549/DDP and H1299/DDP cells transfected with si-NC, si-LHPP-1 and si-LHPP-2 was measured by Western blotting. (**D**) The relative protein expression of E-cadherin, Vimentin, Twist and PCNA in A549/DDP and H1299/DDP cells was measured by Western blotting after silencing of LHPP by si-LHPP. (**E**) The cloning ability of A549/DDP and H1299/DDP cells was determined by colony formation assays after silencing of LHPP by si-LHPP. (**F**) The invasion ability of A549/DDP and H1299/DDP cells was determined by transwell invasion assays after silencing LHPP by si-LHPP. *p < 0.05, **p < 0.01, ***p < 0.001. Colony formation assays were performed in three replicates.

To confirm the role of LHPP in NSCLC/DDP cells, qRT-PCR was carried out to determine LHPP mRNA expression in NSCLC tissues and results demonstrated that LHPP mNRA expression was reduced in these samples (Figure 5A). Subsequently, cells were transfected with si-LHPP-1 or si-LHPP-2 to silence LHPP expression. Both LHPP mRNA and protein expression were silenced by si-LHPP-2 (Figure 5B and 5C, Supplementary Figure 2D). Si-LHPP-2 was used to silence LHPP expression in following experiments. Next, Western blotting was performed to measure EMT markers and showed an increased expression of Vimentin and Twist in NSCLC/DDP cells after silencing LHPP. E-cadherin expression was reduced in NSCLC/DDP cells after silencing LHPP (Figure 5D and Supplementary Figure 2E, 2F). Furthermore, the cloning ability of NSCLC/DDP cells transfected with si-LHPP was enhanced (Figure 5E). The invasion of NSCLC/DDP cells transfected with si-LHPP was also promoted (Figure 5F). These results demonstrated that silencing LHPP promoted EMT progression, proliferation and invasion of NSCLC/DDP cells.

### GAS5 inhibits NSCLC/DDP cell metastasis and epithelial – mesenchymal transition progression

**Figure 6:**
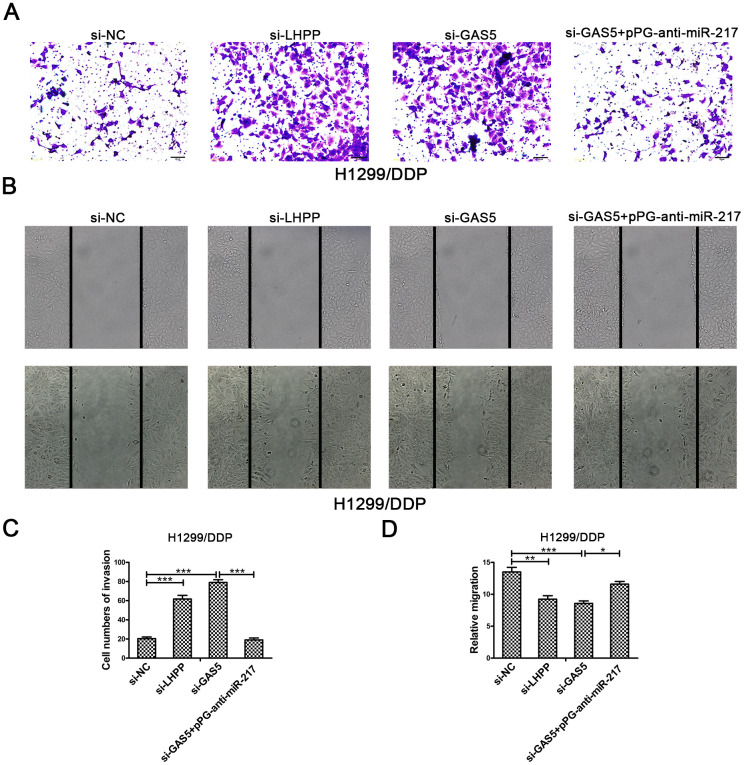
**Silencing LHPP or GAS5 promotes cisplatin-resistant NSCLC cell migration and invasion.** (**A**, **C**) Transwell invasion assays were used to measure the invasion of H1299/DDP cells transfected with si-NC, si-LHPP, si-GAS5 or si-GAS5 + pPG-anti-miR-217. (**B**, **D**) Wound healing assays were used to measure the migration of H1299/DDP cells transfected with si-NC, si-LHPP, si-GAS5 or si-GAS5 + pPG-anti-miR-217. *p < 0.05, **p < 0.01, ***p < 0.001. This experiment was repeated at least three times.

**Figure 7:**
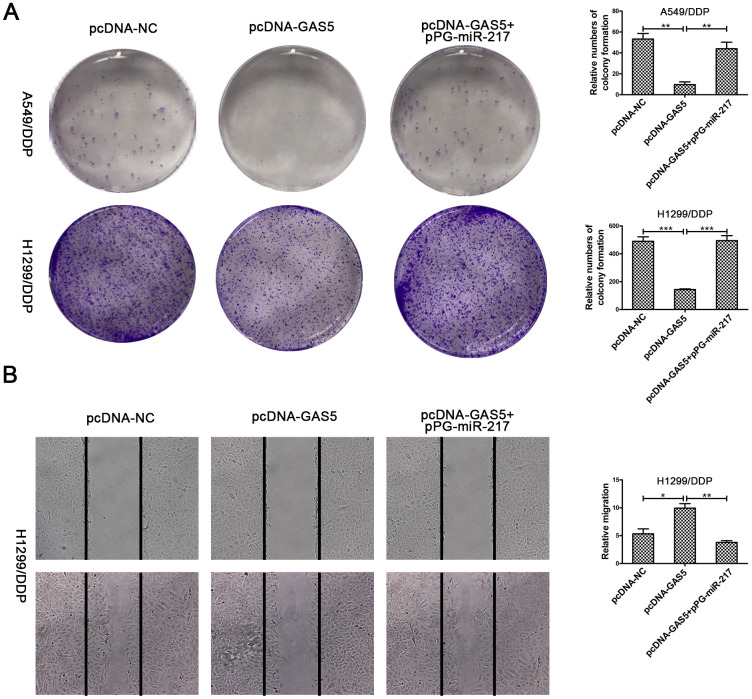
**Upregulation of GAS5 inhibits cisplatin-resistant NSCLC cell cloning ability and migration.** (**A**) Colony formation assays in A549/DDP and H1299/DDP cells transduced with pcDNA-NC, pcDNA-GAS5 or pcDNA-GAS5 + pPG-miR-217 were presented. (**B**) Wound healing assays in H1299/DDP cells transduced with pcDNA-NC, pcDNA-GAS5 or pcDNA-GAS5 + pPG-miR-217 were presented. *p < 0.05, **p < 0.01, ***p < 0.001. Colony formation assays were performed at least three times.

To verify the effect of GAS5 in NSCLC/DDP cell metastasis, transwell invasion assays and wound healing assays were carried out to evaluate the effects of GAS5 knockdown on cell invasion and migration ability. Compared to si-NC, silencing of LHPP and GAS5 in H1299/DDP cells significantly promoted cell invasion, while pPG-anti-miR-217 reversed the effects of si-GAS5 (Figure 6A, 6C). Meanwhile, GAS5 knockdown in H1299/DDP cells remarkedly increased cell migration, and pPG-anti-miR-217 reversed the effect of si-GAS5 (Figure 6B, 6D). Subsequently, colony formation and wound healing assays were aimed to assess the effects of GAS5 overexpression on cell proliferation and migration. We observed that GAS5 overexpression decreased cell proliferation in NSCLC/DDP cells, whereas pPG-miR-217 inhibited this effect (Figure 7A). GAS5 overexpression also resulted in decreased cell migration in H1299/DDP cells and pPG-miR-217 inhibited this effect (Figure 7B).

To explore whether GAS5 inhibits NSCLC/DDP cell metastasis by inducing EMT progression, we carried out immunofluorescence and Western blotting to evaluate the mesenchymal marker expression, Vimentin and the epithelial marker expression, E-cadherin in H1299/DDP cells after GAS5 upregulation. Immunofluorescence results showed that GAS5 overexpression promoted E-cadherin expression and reduced Vimentin expression. MiR-217 had to ability to reverse these observations (Supplementary Figure 3A). Western blotting revealed the same trends (Supplementary Figure 3B, 3C). These results confirmed that GAS5 reduced cisplatin-resistant NSCLC cell metastasis through inducing EMT.

To further confirm that GAS5 represses cell metastasis through LHPP, we analyzed the effects of GAS5 overexpression and LHPP knockdown on NSCLC/DDP cells. As shown in Supplementary Figure 2C, 2D, the invasion of A549/DDP cells was also suppressed by the upregulation of GAS5 and si-LHPP reversed this effect (Supplementary Figure 3D). Meanwhile, GAS5 overexpression reduced Vimentin expression and increased E-cadherin expression in A549/DDP cells (Supplementary Figure 3E, 3F). LHPP silencing promoted Vimentin expression and E-cadherin expression. These results revealed that GAS5 inhibited NSCLC cell metastasis through LHPP.

### GAS5 promoted cisplatin-resistant NSCLC cell proliferation *in vivo*

**Figure 8:**
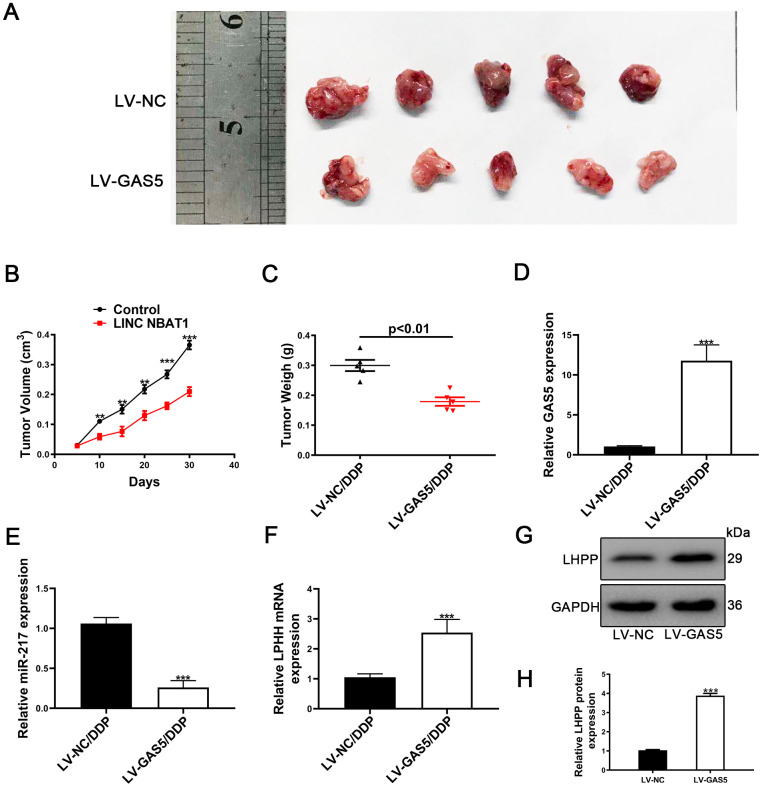
**Upregulation of GAS5 and its influence on tumor growth *in vivo*.** (**A**) Nude mice carrying tumors from A549/DDP/LV-GAS5 and A549/DDP/LV-NC groups were presented. (**B**) Tumor growth curves were calculated every 5 days. (**C**) Tumor weight from A549/DDP/LV-GAS5 and A549/DDP/LV-NC groups was presented. (**D**) The relative expression of GAS5 in tumors from A549/DDP/LV-GAS5 and A549/DDP/LV-NC groups was determined by qRT-PCR. (**E**) The relative expression of miR-217 in tumors from A549/DDP/LV-GAS5 and A549/DDP/LV-NC groups was determined by qRT-PCR. (**F**) The relative mRNA expression of LHPP in tumors from A549/DDP/LV-GAS5 and A549/DDP/LV-NC groups was determined by qRT-PCR. (**G**) The relative protein expression of LHPP in tumors from A549/DDP/LV-GAS5 and A549/DDP/LV-NC groups was determined by Western blotting. *p<0.05, **p<0.01, ***p<0.001. (**H**) Quantitative analysis of LHPP protein expression in A549/DDP and H1299/DDP cells transfected with LV-NC or LV-GAS5 tumor tissue. Tumor xenograft experiments were performed within one-month and other experiments listed were performed in one-week intervals.

To further investigate the role of GAS5 *in vivo*, A549/DDP cells transfected with either LV-NC or LV-GAS5 were subcutaneously injected into nude mice. Compared to control, tumor growth was slower in the LV-GAS5 group (Figure 8A). Tumor volume and tumor weights were also remarkedly smaller in the LV-GAS5 group compared to the LV-NC (Figure 8B, 8C). GAS5 expression was found to be upregulated and miR-217 expression was found to be reduced in tumor tissues containing LV-GAS5 (Figure 8D, 8E). Furthermore, LHPP mRNA and protein expression were measured by using qRT-PCR and Western blotting. It was found that LHPP mRNA and protein expression were also increased in tumor tissues containing LV-GAS5 (Figure 8F, 8G). Altogether, these results demonstrated that GAS5 caused an increased cisplatin-resistant NSCLC cell proliferation both *in vitro* and *in vivo*.

## DISCUSSION

Our study demonstrated that GAS5 expression was reduced in NSCLC/DDP cells. GAS5 was also reduced in NSCLC tissue samples and negatively associated with tumor characteristics. GAS5 overexpression could reduce NSCLC cell cisplatin-resistance as well as metastasis, whereas silencing of GAS5 was capable of promoting NSCLC cell cisplatin-resistance. Importantly, we demonstrated that GAS5 resulted in decreased cisplatin-resistance in NSCLC cells by inhibiting the miR-217/LHPP axis.

LncGAS5 has been regarded as an anticancer gene in many cancers including NSCLC, which can promote the progression of NSCLC [11, 31]. GAS5 expression is significantly reduced in NSCLC tissue samples obtained from patients, and can be used as a high-efficiency biomarker for NSCLC diagnosis [32]. GAS5 has also been reported to repress NSCLC cisplatin-resistance [14, 33]. However, the molecule mechanisms of GAS5 in NSCLC/DDP cell remains unclear. Therefore, we sought to confirm GAS5 role in NSCLC cisplatin-resistance cell. We found that there were low expression levels of GAS5 in NSCLC tissue samples and cisplatin-resistant cell lines. Interestingly, we found that increased GAS5 expression was negatively correlated with clinicopathologic features of NSCLC patients. Importantly, GAS5 expression was reduced in cisplatin-resistant NSCLC cell lines. GAS5 overexpression reduced IC_50_ values of NSCLC/DDP cells and their metastasis through reducing EMT progression. Silencing GAS5 caused the negative effects in NSCLC/DDP cells. Our results confirmed that GAS5 has the ability to impair in NSCLC/DDP cells.

Meanwhile, LHPP could impair NSCLC resistance. LHPP was first isolated in swine brain tissue [15, 16]. LHPP suppressed human HCC and cervical cancer [17, 18]. We found that LHPP knockdown increased the metastasis of DDP NSCLC cells.

Based on our work, we speculated a link between LHPP and GAS5. Salmena et al. [22] first reported that ceRNA forms an extensive regulatory network in the transcriptome, which greatly expands functional genetic information, so ceRNA activity may be an important mechanism for human cancer. Emerging evidence indicated that lncRNAs may interact competitively with endogenous miRNAs by combining the same response elements to regulate target genes.. Therefore, online databases (Starbase, http://starbase.sysu.edu.cn/index.php) were used to search for potential targets of GAS5 and showed that miR-217 may be a potential target. Meanwhile, analysis of the online database (TargetScan, http://www.targetscan.org/vert_72/) was performed to predict that LHPP is a predicted target of miR-217. To confirm the relationship between GAS5 and miR-217, we silenced GAS5 expression in A549/DDP and H1299/DDP cells. GAS5 knockdown improved miR-217 expression and GAS5 overexpression reduced miR-217 expression. Silencing of miR-217 resulted in increased expression of GAS5 and an upregulation of miR-217 caused decreased GAS5 expression. Ago2, a core component of RISC, binds to miRNAs and inhibits the expression of target genes [34]. RIP and miRNA pull-down assays revealed that GAS5 was enriched in beads containing ago2 and can be pull down by biotin-labeled miR-217 in cisplatin-resistant NSCLC cells. The dual-luciferase reporter assay also confirmed that the interaction between GAS5 and miR-217 were functional. These results showed that GAS5 inhibited miR-217 in cisplatin-resistant NSCLC cells.

To verify reciprocity between miR-217 and LHPP, a dual-luciferase reporter assay was performed to confirm the binding between miR-217 and LHPP mRNA. The results showed that combination between miR-217 and LHPP mRNA were functional. Moreover, miR-217 knockdown had an ability to improve LHPP protein expression whereas miR-217 overexpression reduced LHPP expression. GAS5 overexpression increased LHPP expression and GAS5 knockdown showed the opposite effect. It is important to note that miR-217 reversed the positive effects of GAS5 overexpression. Finally, we confirmed that GAS5 inhibited metastasis of cisplatin-resistant NSCLC cells by repressing EMT progression. GAS5 suppressed the growth of NSCLC/DDP tumor *in vivo*. These results showed that GAS5 improved LHPP mRNA and protein expression levels by serving as a ceRNA for miR-217 in cisplatin-resistant NSCLC cell lines.

We performed online database analysis (TargetScan, http://www.targetscan.org/vert_72/) to predict putative targets of miR-217.Figure 3A shows the predicted binding site between miR-217 and LHPP mRNA. Database analysis showed that this binding site (LHPP-wt 5’-AUGCAGU-3’) is the most likely binding area. And our experiments confirmed that miR-217 could combine the 3'-UTR of LHPP mRNA significantly. Due to experimental funding constraints, we did not detect other sites.

**Figure 9:**
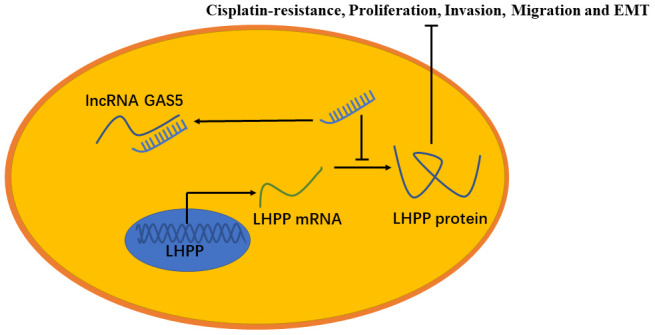
**A model to show the relation between GAS5, miR-217 and LHPP.** GAS5 inhibited NSCLC cisplatin-resistance, metastasis and EMT progression by acting as a miRNA sponge to impair the endogenous effects of miR-217, thereby promoting mRNA and protein expression of LHPP.

In summary, GAS5 inhibited NSCLC cisplatin-resistance, metastasis and EMT progression by impairing the endogenous effects of miR-217, thereby promoting LHPP mRNA and protein expression (Figure 9). The GAS5/miR-217/LHPP regulatory network provides a novel and potential therapeutic target for cisplatin-resistance in NSCLC.

## MATERIALS AND METHODS

### Clinical specimens

Forty-one paired tumor and their tumor adjacent tissues collected between March 2014 to June 2017 in Department of Cardiothoracic Surgery, Xinhua Hospital, Shanghai JiaoTong University School of Medicine (Shanghai, China) were used in this study. Before RNA isolation, each fresh, excised specimen was quickly frozen in liquid nitrogen and stored at -80° C. This study was approved by the Medical Ethics Committee of Xinhua Hospital.

### Cell purchase and culture

H1299 and A549 cell lines were purchased from the American Type Culture Collection (ATCC, Manassas, VA, USA). Incremental increases of cisplatin were added to medium, which established the stable A549/DDP and H1299/DDP. According to previous study [22], the A549 and H1299 cells were treated with incremental increases of cisplatin (Sigma-Aldrich, St. Louis, MO, USA) more than 2 month, starting from 0.5 μg/mL to 8 μg/mL. A549 cell and H1299 cell cisplatin-resistance were confirmed. The A549 and H1299 cells were cultured in RPMI 1640 (Gibco, Carlsbad, USA) medium containing 10% qualified FBS (Thermo Fisher Scientific). The DDP cells were cultured in RPMI 1640 medium supplemented with 10% FBS and 2 μg/mL DDP [35]. Cells were cultured at 37° C in a humidified environment with 5% CO_2_.

### RNAi studies

Two GAS5-small interfering RNAs (si-GAS5), two LHPP-siRNAs (si-LHPP), pPG-miR-217, pPG-anti-miR-217, pPG-miR-NC, were designed and obtained from Sangon Biotech, China. The target sequences for GAS5 small interfering RNAs were [36]: si-GAS5-1: GCCTAACTCAAGCCATTGG, si-GAS5-2: GGTATGGAGAGTCGGCTTG. The target sequences of LPHH-siRNAs were [17]: 5’-CAACCCAAACUGUGUGGUA-3’ for si-LHPP-1, 5’- CAUGAAGGCGCUUGAGUAU-3’ for si-LHPP-2. The pcDNA3.1- lncRNA GAS5 (pcDNA-GAS5) and pcDNA-NC were obtained from GenePharma, China. Cells in six-well plates were not transduced with plasmids or siRNAs by using Lipofectamine 2000 (Invitrogen, USA) seeded until 70% confluency according to the manufacturer’s instruction. Cells were used in further experimentation 48h after transfection.

### Cell viability assays

Cell suspensions (20000 cells / 100 ml) were added in 96-well plates and incubated for 24 hours (37° C, 5% CO_2_). Approximately, each well was added with 10 μL of Cell Counting Kit-8 (CCK-8) solution (Beyotime, China) and incubated for 2h in an incubator after transfection with plasmids or oligonucleotides for various periods (0, 24, 48, 72 and 96 h) or treatments with varying DDP concentrations (2, 4, 8, 12, 16 and 20 μg/mL) for 24h. Cell viability was analyzed using a microplate reader (Molecular Devices, Silicon Valley, California, USA) at a wave length of 450 nm. The formula was listed as follow to calculate the cell growth-inhibition rate: (1 – OD (treatment group) /OD (negative control group)) × 100%. IC_50_ values were then calculated.

### QRT-PCR assalysis

Trizol regent (Beyotime, China) was used to extract total RNA was extracted from tissue samples or cells based on instructions provided by the manufacturer. Subsequently, the one-step RT qPCR SYBRGreen Kit (DBI, Germany) and the ABI 7500 Fast Real-Time PCR System (Life Tech, Carlsbad, California, USA) was used to perform cDNA synthesis and qRT-PCR. For miRNA cDNA synthesis, cDNA of miRNA was obtained by uing miRNA First Strand cDNA Synthesis (Sangon Biotech, Shanghai, China) based on manufacturer’s instruction. 2 μl miRNA RT Enzyme mix, 10 μl 2×miRNA RT Solution mix, 10 μg Total RNA and RNase-free water were mixed together to 20 μl at 4° C. Mix gently and centrifuge for 3~5 s. Warm the reaction mixture at 37° C for 1 h. Then the enzyme was inactivated at 85° C for 5 min and store at 4° C. The relative expression of genes was calculated using the 2^-ΔΔCt^ method, which were normalized to GAPDH and U6. PCR primers were listed in Table 2.

**Table 2:** The PCR primers of gene.

GAS	forward	5’- TGGTTCTGCTCCTGGTAACG-3’
	reverse	5’- AGGATAACAGGTCTGCCTGC-3’
LHPP	forward	5’-CAAACTGTGTGGTAATTGCAGA-3’
	reverse	5-CCAGAGGTCTCCTTGTAGTAAC-3’
MiR-217	forward	5’-ACACTCCAGCTGGGTACTGCATCAGGAACTG-3’
	reverse	5’-TGGTGTCGT GGAGTCG-3’
U6	forward	5’-CGCTTCGGCAGCACATATACTAAAATTGGAAC-3’
	reverse	5’-GCTTCACGAATTTGCGTGTCATCCTTG C-3’
GAPDH	forward	5’- GTCAAGGCTGAGAACGGGAA-3’
	reverse	5’- AAATGAGCCCCAGCCTTCTC-3’

### Western blotting

Cells were lysed by using Radio-Immunoprecipitation Assay (RIPA) buffer supplemented with 10% Phenylmethyl Sulfonylfluoride (PMSF) (Beyotime, China) and total protein was obtained. A total of 30 μg of protein lysate was separated by a 8%-12% gel used in SDS-PAGE (sodium dodecyl sulfate polyacrylamide gel electrophoresis) and then the protein was electrotransferred to a polyvinylidene difluoride (PVDF) membrane. Non-specificity antigen was blocked by 5% not-fat milk and then incubated in primary antibodies overnight at 4° C. Primary antibodies used included: GAPDH (Beyotime, Cat. No. AF5009, China, 1:1000), Vimentin (Proteintech, Cat. No. 60330-1-Ig, USA, 1:1000), E-cadherin (Proteintech, Cat. No. 20874-1-AP, USA, 1:1000), Twist (Proteintech, Cat. No. 25465-1-AP, USA, 1:1000), PCNA (Proteintech, Cat. No. 10205-2-AP, USA, 1:1000) and LHPP (Proteintech, Cat. No. 15759-1-AP, USA, 1:1000). Membranes were washed in 1X TBST thrice and incubated with secondary antibodies (Beyotime, China, 1:2000) for 1h. ECL kit (Millipore, Billerica, MA, USA) was used to visualize the target protein. The endogenous loading control was GAPDH.

### Dual luciferase reporter assays

According to previous study [37], A549/DDP and H1299/DDP cells were co-transduced with pmi-GLO containing putative or mutant 3’-UTRs from GAS5 and LHPP. The pmi-GLO was obtained from Sangon Biotech, China and pRL-TK was obtained from Promega, USA. A549/DDP and H1299/DDP cells was also transfected with pPG-miR-217 or pPG-miR-NC. Dual-luciferase reporter assay kit (Promega, USA) was used to assess the luciferase activities based on the manufacturer’s instruction.

### RNA pull downs

MiR-217, miR-217-mut and NC with biotin (GenePharma, China) were transfected into A549/DDP cells. M-280 streptavidin magnetic beads coated with RNase-free bovine serum albumin and yeast tRNA (Invitrogen, USA) were used to incubate A549/DDP cell lysates at 4° C for 3h. The bound RNAs were purified using TRIzol for the analysis.

### RNA-binding protein immunoprecipitation assays

Approximately 1 × 10^7^ cells were cultured into 10 mm plates and incubated at 37° C until reaching 70% confluence. The EZ-Magna RIP RNA-Binding Protein Immunoprecipitation Kit (Millipore, USA) was used to carry out RNA immunoprecipitation (RIP) assays based on previous study [38]. Normal mouse IgG (Beyotime, China) served as the negative control (NC) and SNRNP70 (Millipore, China) serves as a positive control. Target RNAs were analyzed by qRT-PCR.

### Wound healing assays

Wounds were produced with a 10 μl plastic pipette tip. After creating the wound, cells were cultured in fresh medium supplemented with 1% FBS and 2 μg/mL DDP (Fetal Bovine Serum) for 24h. Take photographs and then assess cell migration.

### Transwell invasion assays

The upper chamber of 24-well transwell plates (Corining, USA) was added with free-serum medium containing 2 μg/mL DDP and 2 × 10^5^ cells. The lower chamber was added with Medium supplemented with 10% FBS and 2 μg/mL DDP. Cells were fixed by 4% paraformaldehyde and then stained with crystal violet. Cotton swab was used to remove the Matrigel layer cells. Count the cells passing through each chamber in five random fields of view under the microscope.

### Colony formation assays

About 1000 A549/DDP and H1299/DDP cells were cultured into each well of six-well plates and incubated in medium supplemented with 10% FBS and 2 μg/mL DDP. After two weeks, cells were fixed with 4% paraformaldehyde for 15 min and then stained with 0.1% crystal violet. The relative numbers of visible colonies were calculated.

### Immunofluorescence analysis

Approximate 1 × 10^6^ A549/DDP and H1299/DDP cells were cultured onto glass coverslips until reaching 50%-70% confluency and then were added with 4% paraformaldehyde. Subsequently, cells were blocked in 5% BSA for 1h. Coverslips were incubated with primary antibodies at 4° C with the following dilutions: E-cadherin (1:100, Abcam, Cat No. ab1416, UK) and Vimentin (1:100, Abcam, Cat No. ab92547, UK). Cells were incubated with secondary antibody (Abcam, UK) for 2h at 37° C. Photos was taken by microscopy (Olympus BX51).

### Lentiviral transfection

LV3-pLVX-GFP-Puro vector containing lncRNA GAS5 (LV-GAS5) or control oligonucleotides (LV-NC) were purchased from GenePharma, China. Lentivirus transfections were performed based on instructions provided by the manufacturer to establish stable GAS5-expressing A549/DDP cells. Control clones were established using similar methodology. LncRNA GAS5 overexpression was confirmed by qPCR.

### Tumor xenografts

Ten 4-week-old male nude mice were randomly divided into two groups (five mice per group). A549/DDP cells (100μl, 1 × 10^6^ cells) that stably expressed GAS5 or NC were subcutaneously injected into nude mice. Tumor volumes were calculated per 5 days. Tumor sizes were using the formula: (mm3) =(L×W2) ×0.5 [39]. After one month, mice were sacrificed and tumors were excise and then weighed. All animal experiments were carried out in the animal laboratory center of Xinhua Hospital. and approved by the Animal Care and Use Committee of Xinhua Hospital.

### Statistical analyses

All data were expressed as mean ± (SD). Normality check was performed and data conforms to normal distribution. A paired sample *t*-test was used to analyze expression the differences of paired sample. The differences between two groups was analyzed by an independent samples *t*-test. The correlated differences about the GAS5 expression in NSCLC patients were analyzed by Pearson’s correlation coefficient. When the P value is less than 0.05, the difference is considered to be statistically significant. ***p < 0.001, **p < 0.01, *p < 0.05. Statistical analysis was performed using SPSS 20.0 (SPSS, USA).

### Availability of data and materials

The datasets used and/or analyzed during the current study are available from the corresponding author upon request.

## Supplementary Material

Supplementary Figures
