# Supplementary material for: The long intergenic noncoding RNA GAS5 reduces cisplatin-resistance in non-small cell lung cancer through the miR-217/LHPP axis
Source: Aging (Albany NY). 2021 Jan 8;13(2):2864–84. doi: 10.18632/aging.202352 (PMC7880381; doi:10.18632/aging.202352)
Supplement: Supplementary Figures [file aging-13-202352-s001.pdf]

SUPPLEMENTARY FIGURES

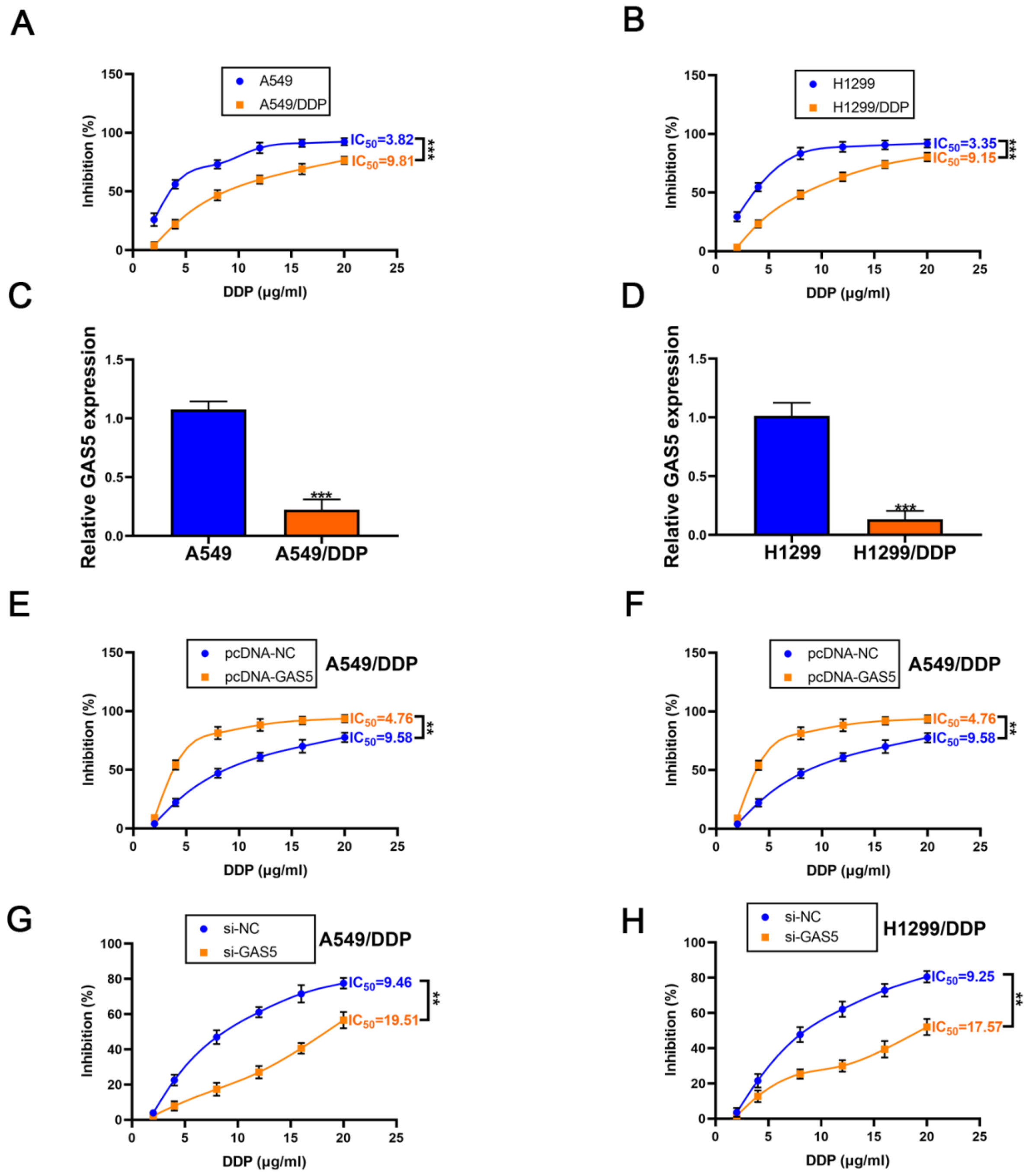

Supplementary Figure 1. LncRNA GAS5 is involved in cisplatin-resistance of NSCLC cells. (**A**, **B**) The sensitivities of cisplatin-resistant NSCLC cells and their parental cells with cisplatin were determined by CCK-8 assays. (**C**, **D**) LncRNA GAS5 expression was detected by qRT-PCR in A549/DDP cells and H1299/DDP cells. (**E**–**H**) The sensitivities of Dox-resistant NSCLC cells under different treatments with Dox were determined by CCK-8 assay. *p<0.05, **p<0.01, ***p<0.001. Cisplatin-resistant cultures were performed over two months and other experiments were performed at least three times within one week.

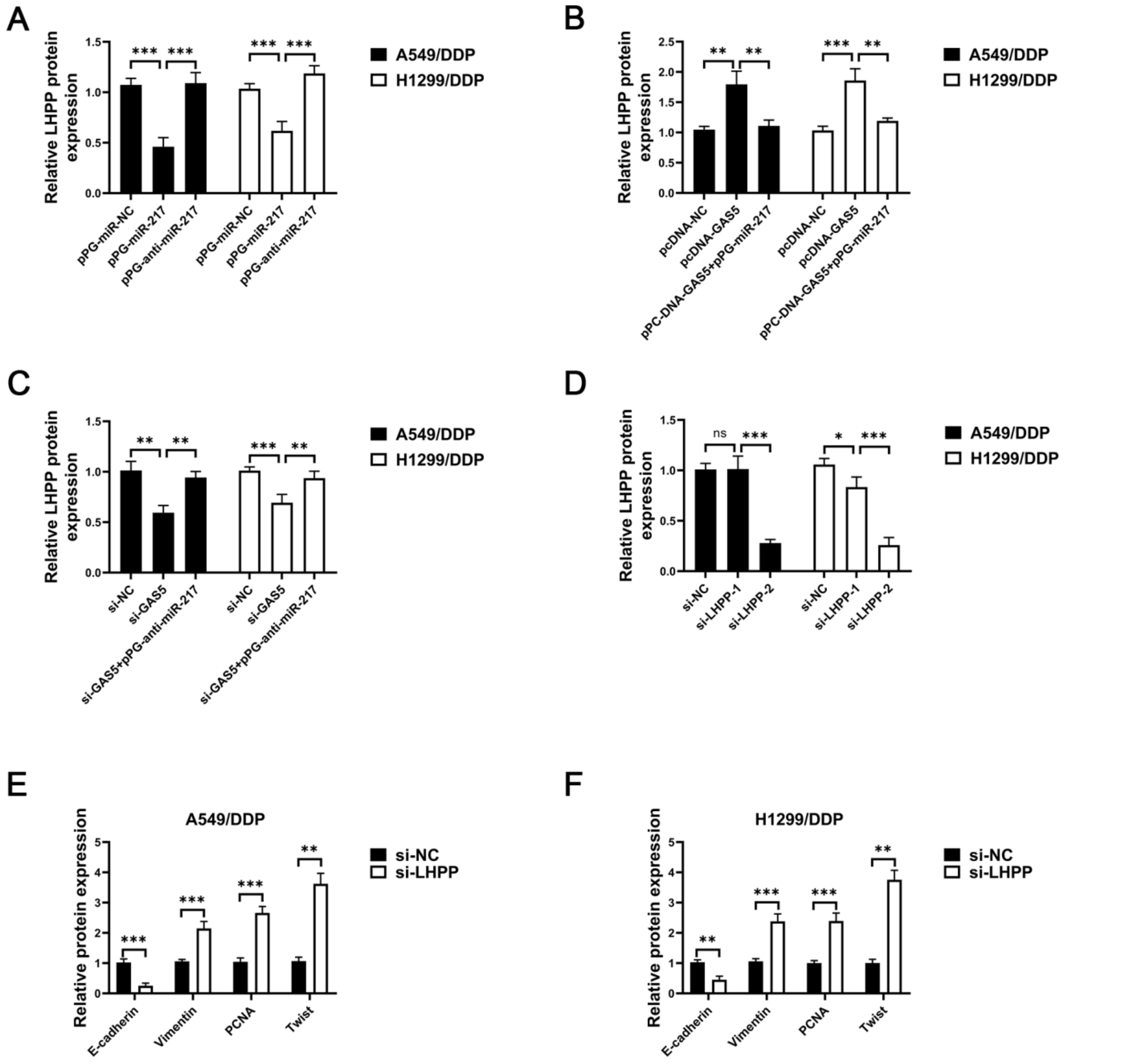

Supplementary Figure 2. Protein bands are quantitated and normalized against their internal controls. (**A**) Quantitative analysis of LHPP protein expression in A549/DDP and H1299/DDP cells transfected with pPG-miR-NC, pPG-miR-217 or pPG- anti-miR- 217. (**B**) Quantitative analysis of LHPP protein expression in A549/DDP and H1299/DDP cells transfected with pcDNA-NC, pcDNA-GAS5 or pcDNA-GAS5 + pPG-miR-217. (**C**) Quantitative analysis for LHPP protein expression in A549/DDP and H1299/DDP cells transfected with si-NC, si-GAS5 or si-GAS5 + pPG-anti-miR-217. (**D**) Quantitative analysis for LHPP protein expression in A549/DDP and H1299/DDP cells transfected with si-NC, si-GAS5-1 or si-GAS5-2. (**E, F**) Quantitative analysis for E-cadherin, Vimentin, PCNA, Twist protein expression in A549/DDP and H1299/DDP cells transfected with si-NC or si-LHPP. *p<0.05, **p<0.01, ***p<0.001.

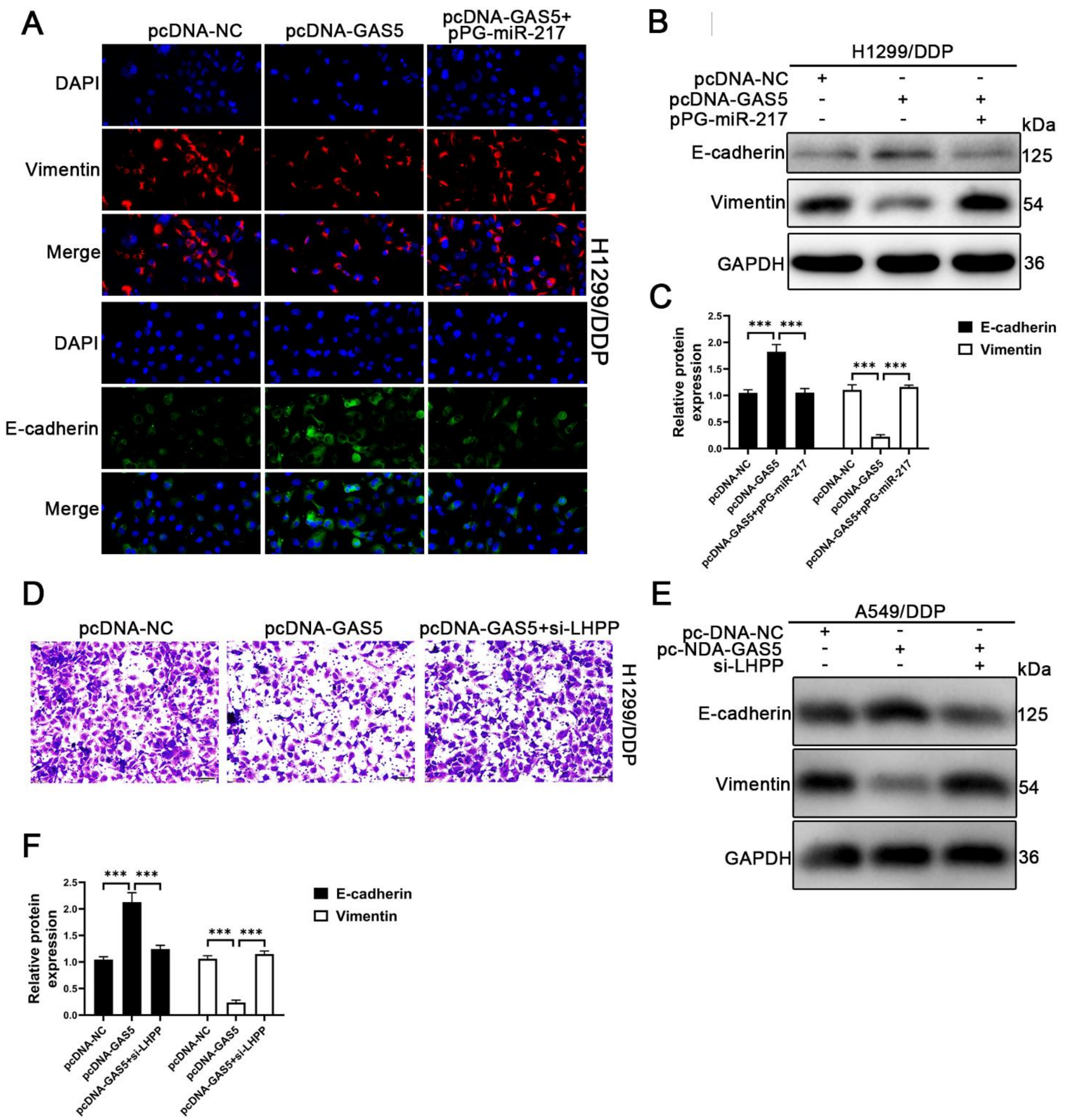

Supplementary Figure 3. Upregulation of GAS5 inhibits NSCLC cell EMT progression. (**A**) E-cadherin and Vimentin expression levels in H1299/DDP transfected with pcDNA-NC, pcDNA-GAS5 or pcDNA-GAS5 + pPG + pPG-miR-217 were detected by immunofluorescence. (**B**) The protein levels of E-cadherin and Vimentin in H1299/DDP cells transfected with pcDNA-NC, pcDNA-GAS5 or pcDNA-GAS5 + pPG-miR-217 were detected by Western blotting. (**C**) Quantitative analysis of E-cadherin and Vimentin protein expression in A549/DDP and H1299/DDP cells transfected with pcDNA-NC, pcDNA-GAS5 or pcDNA-GAS5 + pPG-miR- 217. (**D**) The invasion of A549/DDP cells transfected with pcDNA-NC, pcDNA- lncRNA GAS5 or pcDNA-GAS5 + si-LHPP were determined by transwell assay. (**E**) The protein levels of E-cadherin and Vimentin in H1299/DDP transfected with pcDNA-NC, pcDNA-GAS5 or pcDNA-GAS5 + si-LHPP were detected by Western blotting. (**F**) Quantitative analysis of E-cadherin and Vimentin protein expression in A549/DDP and H1299/DDP cells transfected with pcDNA-NC, pcDNA-GAS5 or pcDNA-GAS5 + si-GAS5. *p<0.05, **p<0.01, ***p<0.001. All experiments were performed at least three times.
